# Education Attainment and Alcohol Binge Drinking: Diminished Returns of Hispanics in Los Angeles

**DOI:** 10.3390/bs9010009

**Published:** 2019-01-14

**Authors:** Shervin Assari, Mehdi Farokhnia, Ritesh Mistry

**Affiliations:** 1 Department of Psychology, University of California, Los Angeles (UCLA), Los Angeles, CA 90095, USA; 2 BRITE Center for Science, Research and Policy, University of California, Los Angeles (UCLA), Los Angeles, CA 90095, USA; 3 Center for Research on Ethnicity, Culture, and Health (CRECH), School of Public Health, University of Michigan, Ann Arbor, MI 48104, USA; 4 Department of Psychiatry, University of Michigan, 4250 Plymouth Rd., Ann Arbor, MI 48109-2700, USA; 5 Section on Clinical Psychoneuroendocrinology and Neuropsychopharmacology, National Institute on Alcohol Abuse and Alcoholism and National Institute on Drug Abuse, National Institutes of Health, Bethesda, MD 20892, USA; mehdi.farokhnia@nih.gov; 6 Department of Health Behavior and Health Education, School of Public Health, University of Michigan, Ann Arbor, MI 48104, USA; riteshm@umich.edu

**Keywords:** socioeconomic status (SES), education attainment, health behaviors, Hispanic, alcohol, binge drinking

## Abstract

According to the minorities’ diminished returns (MDR) theory, socioeconomic status (SES) indicators such as education attainment have smaller protective effects on health risk behaviors for racial and ethnic minority groups in comparison to the ‘dominant’ social group. However, most studies of MDR theory have been on comparison of Blacks versus Whites. Much less is known about diminished returns of SES in ethnic subpopulations (i.e., Hispanics versus non-Hispanic Whites). To test whether MDR also holds for the social patterning of problematic alcohol use among Hispanic and non-Hispanic Whites, this study investigated ethnic variations in the association between education attainment and alcohol binge drinking frequency in a population-based sample of adults. Los Angeles Family and Neighborhood Survey, 2001, included 907 non-Hispanic White and 2117 Hispanic White adults (≥18 years old). Hispanic ethnicity (moderator), education attainment (independent variable), alcohol binge drinking frequency (dependent variable), and gender, age, immigration status, employment status, self-rated health, and history of depression (confounders) were included in four linear regressions. In the overall sample that included both non-Hispanic and Hispanic Whites, higher education attainment was correlated with lower alcohol binge drinking frequency (b = −0.05, 95% CI = −0.09–−0.02), net of covariates. A significant interaction was found between ethnicity and education attainment (b = 0.09; 95% CI = 0.00–0.17), indicating a stronger protective effect of high education attainment against alcohol binge drinking frequency for non-Hispanic than Hispanic Whites. In ethnic-stratified models, higher level of education attainment was associated with lower binge drinking frequency among non-Hispanic Whites (b = −0.11, 95% CI = −0.19–−0.03), but not among Hispanic Whites (b = −0.01, 95% CI = −0.04–0.03). While, overall, higher education attainment is associated with lower frequency of alcohol binge drinking, this protective effect of education attainment seems to be weaker among Hispanic Whites compared to non-Hispanic Whites, a phenomenon consistent with the MDR theory.

## 1. Introduction

Educational attainment, a central socioeconomic resource, is a determinant of health and health behaviors. The literature indicates a protective effect of education attainment on substance use [1], including alcohol use and binge drinking [2]. In the nicotine dependence in teens (NDIT) cohort, compared with those who quit drinking, those who continued drinking were more likely to have no college/university education. Among continued drinkers, those who reduced their binge drinking more quickly had more education attainment [2]. In this paper, we examine whether the association between education attainment and binge drinking depends on ethnicity, i.e., we aim to understand whether Hispanics, an underrepresented ethnic group in the US, experience smaller protective effects of education attainment than non-Hispanics. We focused on binge drinking as it is a major health problem in Whites, including Hispanics.

Socioeconomic status (SES) indicators tend to show smaller effects on health and health behaviors of Blacks versus Whites, a pattern well-described by the minorities’ diminished returns (MDR) theory [3,4]. The effects of education attainment on smoking [5], alcohol use [6], impulse control [7], diet [8], and obesity [9,10] are weaker for Blacks than for Whites. In a 15-year follow up study of economically fragile Black and White families who lived in urban areas, high family SES reduced impulsivity [7], obesity [10], and poor self-rated health [11] for White, but not Black youth. In a national sample of 1500 Black and White adults (65 years or older), the link between education attainment and “ever drinking” was found for Whites, but not Blacks [6].

Although many studies have confirmed the MDR theory [3,4], most of the existing knowledge on this theory is from investigations comparing non-Hispanic Blacks and non-Hispanic Whites. Less is known about how non-Hispanic Whites differ from Hispanic Whites. This is particularly of interest as the Hispanic population is the fastest growing segment of the US population. To our knowledge, only one study to date [12] has compared the effects of SES on health outcomes between Hispanic and non-Hispanic Whites. The study used the Collaborative Psychiatric Epidemiology survey (CPES; 2001 to 2003) data and compared a nationally representative sample of non-Hispanic Whites (*n* = 7587) with Hispanic Whites (*n* = 3620) for the effects of education attainment, income, employment status, and marital status on oral health. Results showed that all SES indicators promoted oral health for non-Hispanic Whites, but not for Hispanic Whites. Results such as these suggest there may be a pattern of disparities in which the historically dominant subgroup (non-Hispanic White) benefit more from the same SES resources than historically marginalized minority populations do. Minorities’ diminished returns (MDR) theory [3,4], an augment to the “Blacks’ diminished returns (BDR)” theory (3), suggests that even among Whites, ethnic minorities would be still at a relative disadvantage compared to non-Hispanics for gaining health benefits from education attainment and other SES resources [12].

It is still unknown whether the MDR theory similarly applies to both race and ethnicity, as society may use different processes to reduce access to and the effects of social and economic resources for various minority groups. This natural extension of the MDR theory allows us to examine important, yet underexplored, relationships to test whether health-related diminished returns of SES resources are specific to Blacks or are observed in other subpopulations. Evidence supporting MDR theory suggests that a key barrier in achieving health equity results from the privilege of Whites, and the solution lies both in the equitable distribution of SES resources and in minimizing the disparate effects of very same SES resources across various subpopulations. In this view, all non-White groups gain less health from their SES resources, as high SES cannot buy Whiteness for non-White groups [13].

### Aim

The current study extends the MDR theory by examining ethnic differences in the association between education attainment and binge drinking frequency in a representative sample of Hispanic and non-Hispanic White adults in Los Angeles.

## 2. Methods

### 2.1. Design and Setting

This study used cross-sectional data from wave 1 of the Los Angeles Family and Neighborhood Survey (L.A.FANS; 2000–2001). L.A.FANS was a representative study of Los Angeles County households [14].

### 2.2. Participants and Sampling

L.A.FANS oversampled low-income families/neighborhoods. The sampling included individuals in 65 census tracts of LA county. We limited our analyses on individuals who (1) were adults, defined as age 18 years or older, (2) had complete data on the model variables, and (3) were either non-Hispanic White or Hispanic White. The current analysis included 907 non-Hispanic White and 2117 Hispanic White adults (age 18 or more).

### 2.3. Study Variables

Variables were selected based on our conceptual model regarding the association between employment status and binge drinking, so they required confounders such as demographic variables (age and gender), immigration status, and other SES (education attainment and marital status). All the study constructs were individual-level variables.

*Main Independent variable.* This study used education attainment as the main independent variable. Participants were asked the following items: “How many grades of school have you finished?” Responses to this question varied between zero to 11. The respondents were also asked “Have you attended college?” and “Have you received a college degree?” Responses were yes and no. The respondents were also asked “What is the highest college or advanced degree you have received?”. Responses included (1) Associate’s/AA, (2) Bachelor’s/BA/BS, (3) Master’s/MA/MS/MBA, and (4) Doctorate/Ph.D. Using all the above items, years of education was operationalized as a continuous variable, with a higher score reflecting higher education attainment.

*Main Dependent Variable*. Binge drinking frequency was assessed using the following single item: In the past 30 days, how many times did you have 5 or more drinks on one occasion? Responses were number of times that could vary between 0 and 60. This variable was operationalized as a continuous variable, with a higher score indicating more binge drinking frequency.

*Confounders.* Our study also included the following sociodemographic variables as confounders. Gender, age, education attainment, employment status, and citizenship status. Age was treated as a continuous measure. Gender was operationalized as a dichotomous measure (1 male, 0 female). Education attainment was number of years of schooling. Employment was a dichotomous variable (1 employed, 0 unemployed or not in the labor market). US citizenship was a dichotomous variable (1 US citizen, 0 not a US citizen). This study also measured self-rated health (SRH) and history of depression as covariates. SRH was measured using the following item: Would you say your health in general is excellent, very good, good, fair or poor? Responses included (1) excellent, (2) very good, (3) good (4) fair, and (5) poor. Lifetime history of depression was self-reported and treated as a dichotomous variable. Has a doctor ever told you that you have depression? Responses were yes and no. Higher SRH reflected worse health.

### 2.4. Ethics

All participants provided written consent. RAND IRB approved the LA-FANS study protocol.

### 2.5. Data Analysis

We analyzed the data using Stata version 15.0 (StataCorp LP, College Station, TX, USA), which allowed us to consider sampling weights. Descriptive statistics, bivariate correlations, and linear regression models were performed. For bivariate correlations, we reported r coefficients from Pearson correlation tests. From our linear regression models, we reported adjusted unstandardized regression coefficients (b) and corresponding 95% confidence intervals (CIs), t and *p* values.

We ran four multivariable linear regression models with binge drinking frequency as the main outcome. *Model 1* and *Model 2* were performed in the pooled sample. *Model 1* did not enter any interaction term; however, *Model 2* did include the ethnicity by education attainment interaction term. *Model 3* and *Model 4* were specified in non-Hispanic Whites and Hispanic Whites, respectively. We tested our main hypothesis in *Model 2* with significance of the interaction term between ethnicity and education attainment.

## 3. Results

### 3.1. Descriptive Statistics

Table 1 describes the study variables in the pooled sample, as well as by ethnicity. Most non-Hispanic Whites were US-born, while most Hispanic Whites were born outside the US. Age (47.68 vs. 37.54; *p* < 0.05) and years of education (14.97 vs. 10.27; *p* < 0.05) were higher in non-Hispanic Whites compared to Hispanic Whites. Binge drinking frequency did not significantly differ between non-Hispanic and Hispanic Whites (0.86 vs. 0.94; *p* > 0.05).

### 3.2. Bivariate Correlations

Table 2 shows three sets of correlation matrices between the study variables; one for the pooled sample and one set for each ethnic group. In the pooled sample, education attainment was not correlated with binge drinking frequency. Education attainment was inversely and significantly correlated with binge drinking frequency among non-Hispanic Whites (r = −0.09; *p* < 0.05), but not among Hispanic Whites (r = −0.01; *p* > 0.05). Employment was positively correlated with binge drinking frequency for non-Hispanic Whites (r = 0.09; *p* < 0.05) and Hispanic Whites (r = 0.09; *p* < 0.05). Males reported more binge drinking frequency, both among non-Hispanic Whites (r = 0.18; *p* < 0.05) and among Hispanic Whites (r = 0.23; *p* < 0.05). Higher age was negatively correlated with binge drinking frequency among non-Hispanic Whites (r = −0.08; *p* < 0.05), but not among Hispanic Whites (r = 0.01; *p* > 0.05). Being a US citizen was positively correlated with binge drinking frequency among non-Hispanic Whites (r = 0.06; *p* < 0.05), but not among Hispanic Whites (r = 0.03; *p* > 0.05).

### 3.3. Linear Regression in the Pooled Sample

Table 3 shows the results of two linear regression models in the pooled sample, with frequency of binge drinking as the main outcome. *Model 1*, which did not include any interaction term, suggested that higher education attainment was associated with lower binge drinking frequency in the pooled sample (b = −0.05, 95% CI = −0.09–−0.02), net of covariates. In this model, ethnicity was not associated with frequency of binge drinking. *Model 2* revealed a significant interaction between ethnicity and education attainment (b = 0.09, 95% CI = 0.00–0.17), suggesting stronger protective effect of education attainment against binge drinking frequency for non-Hispanic Whites than for Hispanic Whites.

### 3.4. Linear Regression by Ethnicity

In ethnic-stratified linear regression models, education attainment was inversely associated with binge drinking frequency among non-Hispanic Whites (b = −0.11, 95% CI = −0.19–−0.03) (*Model 3*) but not among Hispanic Whites (b = −0.01, 95% CI = −0.04–0.03) (*Model 4)*. For non-Hispanic Whites, in addition to education attainment, US citizenship, male gender, and employment status also predicted binge drinking frequency. For Hispanic Whites, male gender was the only predictor of binge drinking frequency (Table 4).

## 4. Discussion

In the present study, equal education attainment was unequally associated with binge drinking frequency among Hispanic and non-Hispanic White adults. Our analyses showed that among Whites, Hispanics are at a relative disadvantage compared to non-Hispanics for gaining a protective effect of their education attainment in terms of lower frequency of binge drinking.

The findings of this study extend the research on the diminished returns of SES resources based on race [3,4] by examining ethnicity. To date, the majority of investigations on diminished returns have focused on comparison of non-Hispanic Blacks and non-Hispanic Whites, with less research available on the role of Hispanic ethnicity. This study showed that even among Whites, ethnicity modifies the benefits gained from SES resources (i.e. the protective effect of education attainment on problematic alcohol use).

Similar patterns are shown for other health outcomes in comparison of non-Hispanic Whites and Blacks. The effects of education attainment [15] and employment [16] on mortality are stronger for non-Hispanic Whites than for non-Hispanic Blacks. Similarly, both education attainment [17] and income [18,19,20] better reduce risk of depression for non-Hispanic Whites than non-Hispanic Blacks.

Discrimination has been shown to reduce the health gain of SES [21]. The diminished returns of education attainment among minorities in the US have been attributed to the existing racism and discrimination. As SES of non-Whites increases, they become in more contact with Whites [19,22], which in turn increases their exposure to interpersonal discrimination [23,24]. For example, high SES adolescent minorities more frequently attend predominantly White schools [19] and high SES adult minorities are more likely to work in predominantly White work places [22].

In addition to interpersonal discrimination that is mainly due to contact with other racial and/or ethnic groups [23,24], the preferences and practices of the labor market also play significant roles in reducing minorities’ health gains from SES factors, particularly from education attainment [25]. Due to the preferences of labor market, the very same education attainment generates more income for Whites than for other groups [26], a pattern which also explains why employment generates more life expectancy for non-Hispanic Whites than non-Whites [16]. High education attainment better helps White families than non-White families to escape poverty [27]. In a 10-year longitudinal study, higher levels of education attainment predicted a larger increase in income for non-Hispanic Whites but not for non-Hispanic Blacks [26]. At the same time, neither education attainment nor income were associated with a positive affect for non-Hispanic Blacks, while both of these SES indicators resulted in a positive affect for Whites [26]. In a study using data from the Health Information National Trends Survey (HINTS), income mediated the diminished returns of education attainment on self-rated health for non-Hispanic Blacks compared to non-Hispanic Whites [28]. The case of minorities’ diminished returns (relative to Whites) is extreme for non-Hispanic Black men. In cross-sectional studies, highly educated and high-income Black men are found to be at an increased risk of depression [20,29]. In a longitudinal study of a representative sample, non-Hispanic Black men experienced an increased risk of depressive symptoms over time [17]. It has also been shown that non-Hispanic Black boys from high-income families are at an increased risk of depression [18]. Most educated non-Hispanic Black women are also at a higher risk of suicidal ideation [30]. In another study on a representative sample of adults from Michigan, high income was associated with better self-rated mental health of non-Hispanic Whites, but not non-Hispanic Blacks [31].

Amongst Whites, highly educated Hispanics are at a higher risk of frequent alcohol binge drinking compared to non-Hispanics, possibly because upward social mobility is more stressful for ethnic minority groups [21]. Even for the families and individuals who successfully climb the social ladder, upward social mobility (e.g., high education attainment and income) is not similarly rewarded in terms of life conditions, income, purchasing power, and health [3,4].

The weaker effects of education attainment in reducing high risk behaviors such as alcohol binge drinking in minorities may explain why SES indicators such as education attainment better reduce the risk of chronic medical disease [32] and depression [17,33] in racial/ethnic majority versus minority groups. Excessive alcohol/substance use is a risk factor for chronic medical diseases [34] and depression [35]. For example, binge drinking increases the risk of metabolic disorders, such as obesity, diabetes, and cardiovascular diseases [34]. Future research should test whether the diminished returns of SES on health behaviors may explain the diminished returns of SES on depression and other chronic diseases for racial/ethnic minority populations.

### 4.1. Implications

The present results suggest that among non-Hispanic Whites, high-SES individuals are at a lower risk of frequent alcohol binge drinking compared to low-SES individuals. This SES effect is absent for high-SES Hispanic Whites, who are at high risk of binge drinking. Subsequently, higher education attainment and SES should not reduce the probability of screening, diagnosis, and treatment of problematic alcohol drinking for Hispanic Whites. Often, high SES is wrongly assumed to imply low risk for health behavioral risks [36]; even if racial and ethnic minorities achieve a higher level of SES, they do not always reap the health benefits. While higher SES may indicate a lower need for prevention, education, and treatment of problem drinking and alcohol-related problems for non-Hispanic Whites, this is not the case for Hispanic Whites. It appears the interventions for minority populations like Hispanics should be designed to not only reach low SES groups, but more broadly to reach even high SES groups.

### 4.2. Limitations

The current study had a number of limitations. First, the cross-sectional design of the study limits any causal inferences. While low SES may lead to problematic alcohol use, excessive alcohol drinking and traits associated with it may also cause downward social mobility and low SES. The reverse causation is, however, more relevant to SES indicators that are more likely to change later in life (e.g., income, marital status, and employment) compared to education attainment, which may increase over time, but never decreases. Second, the sample size was not balanced between our ethnic groups, with a lower number of non-Hispanic Whites being part of this study. Differential sample size results in differential statistical power, especially for ethnic-stratified models. However, this point was not a concern in our study, because the association of interest was not statistically significant in the group with a larger sample size. Third, the two ethnic groups were not balanced in terms of age, which may have implications for the association between SES and drinking behaviors. Fourth, the outcome in this study was frequency of alcohol binge drinking, and not a documented alcohol use disorder (diagnosed by a psychiatrist or based on a structured clinical interview). Fifth, a limited number of confounders were included in the statistical models. Several contextual factors, such as availability of alcohol outlets (e.g., liquor stores and corner stores), as well as density of job opportunities, SES of the area, and neighborhood safety, may confound the association between personal SES and the pattern of alcohol use by race/ethnicity [37]. In addition to contextual factors, behavioral coping may also play a role in shaping drug-seeking behaviors among ethnic minorities, as substance and alcohol may be used to cope with discrimination, financial difficulty, unemployment, or food insecurity [38,39]. Although we controlled for immigration status, we did not measure acculturation and duration of being a resident of the US which may have significant effects on drinking behaviors, including binging. Sixth, self-report measures of binge drinking frequency may be prone to different levels of measurement error across ethnic groups. We cannot rule out the possibility of ethnic differences in validity and accuracy of self-reported binge drinking data [40]. Seventh, gender may alter the correlates of problematic alcohol use across ethnic minority groups. Future research should test whether minorities’ diminished returns differ for males and females [6,41,42]. In addition, we do not know whether highly educated Hispanics more frequently visit social settings where alcohol is consumed than highly educated non-Hispanic Whites or not. Last but not least, Hispanic Whites are a heterogeneous group of people, with different socioeconomic, cultural, and historical backgrounds. Similar studies should be conducted based on country of origin as well as immigration status. Substance use patterns differ among Hispanics based on individuals’ country of origin and ethnic heritage [43]. There is a need for replication of our findings using prospective longitudinal design, including other SES indicators, other age groups, and using more robust outcomes, such as physician diagnosis of alcohol use disorder. It is particularly important to study alcohol use disorder (AUD), which indicates chronic excessive use of alcohol. There is also a need to study other substances. Depending on the distribution of the outcomes, future attempts may treat their outcomes as a count variable instead, using Poisson regression. As there is an interplay between education, poverty, and employment, future analytical models may allow the reciprocal effects between various SES indicators.

### 4.3. Conclusions

To conclude, while overall, higher education attainment is associated with a lower frequency of alcohol binge drinking, this protective effect may be absent or weaker for Hispanic Whites compared to non-Hispanic Whites. This finding suggests that MDR theory applies to epidemiological studies of social patterning of alcohol-related behaviors in ethnically diverse populations.

## Figures and Tables

**Table 1:** Descriptive statistics in the pooled sample and by ethnicity.

Characteristics	Pooled Sample (*n* = 3024)			Non-Hispanic Whites (*n* = 907)			Hispanic Whites (*n* = 2117)		
	*%*	*SE*	*95% CI*	*%*	*SE*	*95% CI*	*%*	*SE*	*95% CI*
Ethnicity									
Non-Hispanic White	49.06	0.02	45.99 to 52.13	-	-	-	-	-	-
Hispanic White	50.94	0.02	47.87 to 54.01	-	-	-	-	-	-
US Citizen *									
No	30.40	0.01	27.92–33.00	5.07	0.01	3.41–7.47	54.80	0.02	51.15–58.39
Yes	69.60	0.01	67.00–72.08	94.93	0.01	92.53–96.59	45.20	0.02	41.61–48.85
Gender									
Female	50.59	0.02	47.53–53.64	50.66	0.03	45.69–55.62	50.52	0.02	46.90–54.12
Male	49.41	0.02	46.36–52.47	49.34	0.03	44.38–54.31	49.48	0.02	45.88–53.10
Depression (Self-Report) *									
No	93.69	0.01	91.87–95.12	90.98	0.02	87.55–93.53	96.30	0.01	94.69–97.43
Yes	6.31	0.01	4.88–8.13	9.02	0.02	6.47–12.45	3.70	0.01	2.57–5.31
Employed									
No	33.65	0.01	30.81–36.61	35.37	0.02	30.73–40.32	31.98	0.02	28.79–35.36
Yes	66.35	0.01	63.39–69.19	64.63	0.02	59.68–69.27	68.02	0.02	64.64–71.21
	***Mean***	***SE***	***95% CI***	***Mean***	***SE***	***95% CI***	***Mean***	***SE***	***95% CI***
Age *	42.58	0.60	41.40–43.76	47.68	0.98	45.76–49.60	37.54	0.61	36.34–38.74
Self-Rated Health (Poor)	2.49	0.04	2.42–2.56	2.30	0.06	2.19–2.41	2.68	0.04	2.59–2.76
Education (Years) *	12.61	0.13	12.35–12.87	14.97	0.14	14.69–15.26	10.27	0.17	9.94–10.60
Binge Drinking Frequency *	0.80	0.10	0.60–1.00	0.86	0.17	0.52–1.20	0.74	0.11	0.53–0.96

* *p* < 0.05 for comparison of Non-Hispanic and Hispanic Whites.

**Table 2:** Correlation matrix in the pooled sample and by ethnicity.

Characteristics	1	2	3	4	5	6	7	8	9
***Pooled Sample* (*n* = 3024)**									
1 Ethnicity (Hispanic)	1.00 *								
2 US citizen	−0.50 *	1.00 *							
3 Male	−0.06 *	0.03	1.00 *						
4 Age	−0.26 *	0.24 *	0.04 *	1.00 *					
5 Self-Rated Health (SRH) (Poor)	0.26 *	−0.24 *	−0.11 *	0.12 *	1.00 *				
6 Depression (Self Report)	−0.08 *	0.10 *	−0.06 *	0.10 *	0.18 *	1.00 *			
7 Employed	−0.07 *	0.13 *	0.28 *	−0.10 *	−0.18 *	−0.09 *	1.00 *		
8 Education (years)	−0.55 *	0.52 *	0.03	0.02	−0.37 *	0.03	0.20 *	1.00 *	
9 Binge Drinking Frequency	−0.01	0.04 *	0.22 *	−0.02	−0.01	0.01	0.09 *	−0.01	1.00 *
***Non-Hispanic Whites* (*n* = 907)**									
2 US citizen	-	1.00 *							
3 Male	-	0.05 *	1.00 *						
4 Age	-	0.13 *	0.02	1.00 *					
5 SRH (Poor)	-	−0.03	−0.07 *	0.19 *	1.00 *				
6 Depression (Self Report)	-	0.07 *	−0.08 *	0.03	0.29 *	1.00 *			
7 Employed	-	0.08 *	0.19 *	−0.23 *	−0.22 *	−0.16 *	1.00 *		
8 Education (years)	-	−0.01	0.02	0.04 *	−0.25 *	−0.07 *	0.16 *	1.00 *	
9 Binge Drinking Frequency	-	0.06 *	0.18 *	−0.08 *	0.00	0.00	0.09 *	−0.09 *	1.00 *
***Hispanic Whites* (*n* = 2117)**									
2 US citizen	-	1.00 *							
3 Male	-	−0.02	1.00 *						
4 Age	-	0.14 *	0.03	1.00 *					
5 SRH (Poor)	-	−0.16 *	−0.10 *	0.21 *	1.00 *				
6 Depression (Self Report)	-	0.08 *	−0.06 *	0.11 *	0.16 *	1.00 *			
7 Employed	-	0.13 *	0.31 *	−0.08 *	−0.15 *	−0.07 *	1.00 *		
8 Education (years)	-	0.40 *	0.00	−0.23 *	−0.29 *	0.00	0.20 *	1.00 *	
9 Binge Drinking Frequency	-	0.03	0.23 *	0.01	−0.01	0.01	0.09 *	−0.01	1.00 *

* *p* < 0.05 (Pearson Correlation Test).

**Table 3:** Results of two linear regression models in the pooled sample.

Characteristics	*b*	*SE*	*95% CI*	t	*p*
***Model 1 in All* (*n* = 3024)**					
Ethnicity (Hispanic)	−0.25	0.30	−0.84–0.35	−0.81	0.415
US citizen	0.62	0.24	0.15–1.08	2.61	0.009
Age	−0.02	0.01	−0.03–0.00	−2.23	0.026
Male	1.05	0.20	0.67–1.43	5.38	0.000
SRH (Poor)	0.03	0.13	−0.21–0.28	0.27	0.790
Depression (Self Report)	0.21	0.34	−0.46–0.89	0.63	0.531
Employed	0.38	0.16	0.06–0.69	2.36	0.018
Education Attainment (years)	−0.05	0.02	−0.09–−0.02	−2.76	0.006
Intercept	1.06	0.38	0.31–1.81	2.76	0.006
***Model 2 in All* (*n* = 3024)**					
Ethnicity (Hispanic)	−1.42	0.81	−3.00–0.17	−1.75	0.080
US citizen	0.52	0.23	0.08–0.96	2.32	0.020
Age	−0.02	0.01	−0.03–0.00	−2.15	0.032
Male	1.06	0.20	0.68–1.45	5.38	<0.001
SRH (Poor)	0.03	0.12	−0.22–0.27	0.22	0.828
Depression (Self Report)	0.21	0.34	−0.46–0.88	0.61	0.540
Employed	0.39	0.16	0.07–0.70	2.40	0.017
Education Attainment (years)	−0.11	0.04	−0.20–−0.02	−2.46	0.014
Education Attainment (years) × Ethnicity (Hispanic)	0.09	0.04	0.00–0.17	1.90	0.050
Intercept	1.92	0.73	0.49–3.36	2.62	0.009

Notes: b = unstandardized adjusted regression coefficient, SE = Standard Error. Model 1 only includes the main effects. Model 2 also includes an interaction between ethnicity and education attainment.

**Table 4:** Results of two linear regression models by ethnicity.

Characteristics	*b*	*SE*	*95% CI*	t	*p*
***Model 3 in Non-Hispanic Whites (n* = 907)**					
US citizen	0.78	0.29	0.21–1.34	2.68	0.007
Age	−0.02	0.01	−0.04–0.00	−1.65	0.099
Male	1.12	0.32	0.50–1.73	3.54	<0.001
SRH (Poor)	0.12	0.25	−0.37–0.60	0.47	0.637
Depression (Self Report)	0.29	0.52	−0.74–1.31	0.55	0.585
Employed	0.74	0.24	0.27–1.20	3.09	0.002
Education (years)	−0.11	0.04	−0.19–−0.03	−2.56	0.011
Intercept	1.34	0.69	−0.01–2.69	1.94	0.052
***Model 4 in Hispanic Whites (n* = 2117)**					
US citizen	0.35	0.27	−0.17–0.87	1.31	0.189
Age	−0.01	0.01	−0.02–0.01	−1.10	0.273
Male	1.04	0.25	0.56–1.53	4.24	<0.001
SRH (Poor)	−0.04	0.09	−0.21–0.14	−0.44	0.659
Depression (Self Report)	−0.04	0.24	−0.51–0.43	−0.16	0.873
Employed	0.03	0.24	−0.43–0.49	0.14	0.891
Education (years)	−0.01	0.02	−0.04–0.03	−0.44	0.660
Intercept	0.51	0.42	−0.30–1.33	1.23	0.217

B = unstandardized adjusted regression coefficient, SE = Standard Error.
